# Early Predictors for Infectious Complications in Patients With Spontaneous Intracerebral Hemorrhage and Their Impact on Outcome

**DOI:** 10.3389/fneur.2019.00817

**Published:** 2019-08-06

**Authors:** Anna Lindner, Mario Kofler, Verena Rass, Bogdan Ianosi, Max Gaasch, Alois J. Schiefecker, Ronny Beer, Sebastian Loveys, Paul Rhomberg, Bettina Pfausler, Claudius Thomé, Erich Schmutzhard, Raimund Helbok

**Affiliations:** ^1^Neurological Intensive Care Unit, Department of Neurology, Medical University of Innsbruck, Innsbruck, Austria; ^2^Institute of Medical Informatics, University for Health Sciences, Medical Informatics and Technology, Hall, Austria; ^3^Department of Neuroradiology, Medical University of Innsbruck, Innsbruck, Austria; ^4^Department of Neurosurgery, Medical University of Innsbruck, Innsbruck, Austria

**Keywords:** intracerebral hemorrhage, risk factors, infections, critical care, neurology, infectious complications

## Abstract

**Background:** Infectious complications (IC) commonly occur in patients with intracerebral hemorrhage (ICH) and are associated with increased length of hospitalization (LOS) and poor long-term outcome. Little is known about early ICH-related predictors for the development of IC to allow appropriate allocation of resources and timely initiation of preventive measures.

**Methods:** We prospectively enrolled 229 consecutive patients with non-traumatic ICH admitted to the neurocritical care unit (NICU) of a tertiary care hospital. Patients were screened daily for IC. Multivariable regression models using generalized linear models were used to identify associated factors with the occurrence of IC and to study their impact on functional outcome, which was assessed using the modified Rankin Scale Score (mRS) after 3 months. Unfavorable outcome was defined as mRS ≥3.

**Results:** The most common IC were pneumonia (*n* = 64, 28%) and urinary tract infection (*n* = 54, 24%), followed by sepsis (*n* = 9, 4%) and ventriculitis (*n* = 4, 2%). Patients with a higher admission ICH Score (>2) had higher odds to develop any IC during NICU stay (OR = 1.7, 95% CI 1.2–2.3, *p* = 0.02). Moreover, early-onset pneumonia (≤48 h after admission) was predictive of sepsis occurring at a later time-point (median at day 11 [IQR = 6–34 days], adjOR = 22.5, 95% CI 4.88–103.6, *p* < 0.001). Having at least one IC and pneumonia itself were independently associated with unfavorable 3-months outcome (adjOR = 3.0, 95% CI 1.41–6.54, *p* = 0.005; adjOR = 4.2, 95% CI 1.33–13.19, *p* = 0.015, respectively). All patients with sepsis died or had poor functional outcome.

**Conclusions:** Infectious complications are common in ICH patients and independently associated with unfavorable outcome. An ICH Score >2 on admission and early pneumonia may help to early identify patients at high risk of IC to allocate resources and start careful surveillance.

## Introduction

Intracerebral hemorrhage (ICH) accounts for 9–27% of all strokes worldwide and is associated with high rates of disability and mortality (1). Approximately 40% of ICH patients die within 30 days and 55% within the first year (2). The majority of surviving patients are functionally disabled with only 12–39% reaching long-term functional independence (3, 4). Besides the primary brain damage secondary complications largely contribute to poor functional outcome (5, 6). Consequently, the prevention and adequate treatment of secondary complications, including infections, together with early risk stratification of these patients is of high interest (7). During NICU stay up to 70% of ICH patients are known to develop at least one complication (8) and 23–58% of patients may suffer from infectious complications (IC) (7, 9–12). While it is well-known that infections complicate hospital course and are associated with poor functional outcome, little is known about their chronological occurrence and their cumulative effect on outcome. Furthermore, it is not well-described if early infections trigger late infectious complications. The ICH Score (13) has recently been shown to be the most extensively validated severity score in ICH patients (14) and is commonly used in clinical practice to stratify mortality risk after ICH. However, its role in the prediction of infectious complications is unclear.

In this study, we intended [1] to investigate the chronological course of IC, [2] to identify early, potentially modifiable predictors including the ICH Score for the development of infections after ICH, and [3] to investigate whether early infections are predictive of later complications. Moreover, we aimed to [4] quantify the impact of IC on the length of NICU stay and functional outcome. We hypothesized that the ICH Score is a useful tool in IC prediction and that IC are associated with poor functional outcome.

## Methods

### Patient Selection and Care

**Figure 1:**
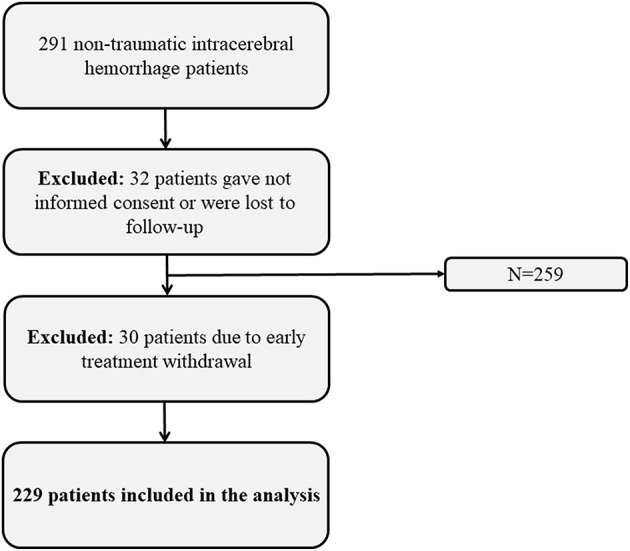
Flow chart showing the selection of eligible patients.

Two hundred ninety-one consecutive patients with non-traumatic ICH admitted to the neurological ICU (NICU) at a tertiary care hospital between 2011 and 2016 were prospectively enrolled. Inclusion criteria were: (a) spontaneous ICH diagnosed on computed tomography (CT) or magnetic resonance imaging (MRI), (b) age > 18 years, (c) written informed consent according to local regulations in accordance with the declaration of Helsinki, and (d) completed follow-up at 3 months. 32/291 patients did not give consent to participate or were lost to follow-up. Another 30/291 patients were excluded because of early treatment withdrawal, leaving 229 patients eligible for the current analysis. Withdrawal of care was decided following the criteria of age >80 years, limited life expectancy due to comorbidities and massive ICH with clinical or radiographic signs of herniation (Figure 1). Approval for the study was granted by the hospital's institutional review board (Medical University Innsbruck, AN4088292/4.3).

### Clinical Management, Data Collection, Clinical and Radiographic Variables and Outcome

Following the local policy all patients diagnosed with an ICH are primarily admitted to an ICU. Less than 3% of patients were admitted to the stroke unit due to lack of available beds in the NICU during the study period. Patients' and disease specific characteristics, treatment interventions, hospital complications and outcomes were prospectively recorded in the institutional ICH database during weekly meetings held by the study team and treating neurointensivists. The Glasgow Coma Scale Score (GCS), the Acute Physiology And Chronic Health Evaluation (APACHE)—II (15), and the ICH Score (13) on admission, were used for clinical and radiographic grading. Clinical management conformed to American Heart Association and European Stroke association guidelines (5, 16). Vital signs and laboratory data were saved in a patient data management system (PDMS) (Centricity™ Critical Care 8.1 SP7; GE Healthcare Information Technologies, Germany).

Early neurological deterioration was defined as ≥2 points decrease in GCS or a new focal finding occurring within a 24 h period after admission or in the early postoperative phase. Mechanical ventilation, nasogastric tube feeding, hydrocephalus requiring external ventricular drain (EVD) placement and hematoma evacuation were determined as hospital-related procedures. Survival and functional outcome was assessed at 3 months after ictus by a study nurse blinded to the clinical course of the patient using the modified Rankin Scale Score (mRS) (17), with unfavorable outcome defined as mRS ≥3.

### Definitions of Infectious Complications

Infectious complications were prospectively confirmed in a weekly study meeting and the date of onset was recorded, according to predefined criteria set forth by the Centers for Disease Control and Prevention criteria (CDC-criteria) (18), and the American Thoracic Society (19). Pneumonia was diagnosed in patients who had a new or progressive pulmonary infiltrate with clinical evidence of an infectious origin (two out of three: new onset of fever (≥38.3°C), leukocytosis (>12,000/mm^3^) or leukopenia (<4,000/mm^3^), purulent sputum). Pneumonia diagnosed within 48 h after admission was defined as early onset pneumonia, whereas pneumonia occurring >48 h after initiation of mechanical ventilation was defined as ventilator associated pneumonia (VAP) (19). Urinary tract infection (UTI) was diagnosed in patients with a positive microbiological culture of urine (10^5^ colony forming units (CFU)/mL) with a maximum of two species of organisms and the occurrence of one of the following symptoms: fever (≥38.3°C), high frequency, urgency, dysuria, or suprapubic tenderness. Sepsis was defined according to the CDC-criteria (18) by either [1] isolation of pathogenic organisms in the blood culture, which could not be related to an infection at another location or [2] at least one of the following three clinical conditions: fever (≥38.3°C), chills, or hypotension (systolic blood pressure <100 mmHg) and any of the following: (a) common skin contaminant isolated from two blood cultures drawn on separate occasions, (b) common skin contaminant isolated from patient's blood culture with intravascular access device and physician institutes appropriate antimicrobial therapy, or (c) positive antigen test on blood and organism is not related to infection at another site. Ventriculitis was defined according to the CDC-criteria: presentation of pathogens in cerebrospinal fluid (CSF) culture, or in patients who had one of these conditions: fever (≥38.3°C), headache, meningism or cranial nerve signs, and at least one of the following findings in the CSF analysis: an increase in leukocytes, elevated protein and/or decreased glucose, positive culture, a positive antigen detection, detection of pathogens or antigens in the blood or an increased diagnostic single antibody titer (18). If the same infectious complication occurred twice during NICU stay, only the first occurrence was rated. First line antimicrobial treatment included ampicillin/sulbactam, doxycycline, or fluoroquinolones, followed by second line escalation therapy including aminopenicillin (piperacillin/tazobactam) or macrolide antibiotics. Third line treatments included vancomycin or linezolid against gram-positive rods and meropenem or cefepime for resistant gram-negative rods.

### Statistical Analysis

Statistical analysis was performed using IBM-SPSS V24.0 (SPSS Inc, Armonk, NY). Continuous variables were assessed for normality using Shapiro-Wilk test. Values were reported as median and interquartile range [IQR]. We performed univariate analysis to identify clinically relevant risk factors for every type of IC using generalized linear models with a binary logistic function. Significantly associated factors (*p* < 0.1) in univariate analysis were included stepwise in multivariable logistic regression models using generalized linear models with a binary logistic function and Logit as link function and retained if significant (*p* < 0.05). The ICH Score was kept in all models as ordinal independent variable irrespective of its significance in order to account for disease severity. Factors associated with poor functional outcome were identified using the same approach and entered in multivariable generalized linear models. Of note, except the infratentorial hemorrhage location, all subcategories of the ICH Score were associated with poor outcome in univariate analysis.

ROC (receiver operating characteristic) curves were calculated for the ICH Score and each subcategory with the development of infectious complications as categorical variable. AUC for the ICH Score and GCS on admission were similar, however, based on the best model of fit (Akaike's Information Criterion, AIC), the predictive value of the ICH Score was significantly better as compared to the admission GCS Score. Moreover, a cox proportional-hazards regression was used, and the ICH Score was dichotomized at >2 and ≤2 based on the median. Furthermore, the median revealed the optimal cut-off point of the ICH Score for IC prediction evaluated by the Youden index to find the maximal sum of sensitivity and specificity. Statistical significance was judged to a *p*-value <0.05.

## Results

### General Characteristics

Patient characteristics, hospital complications and outcomes are presented in Table 1. Sixty-three patients/229 (28%) were comatose on admission. Hematoma location was supratentorial in 206 patients (90%) [cortical: 78 (34%), subcortical white matter: 39 (17%), basal ganglia: 55 (24%), thalamic: 34 (15%)] and infratentorial in 26 patients (10%) [cerebellum: 13 (6%), brainstem: 7 (3%)]. Fifty-six patients (25%) underwent hematoma evacuation within 24 h after admission. In-hospital mortality was 26% (*n* = 60).

**Table 1:** Baseline characteristics, complications, and outcome.

	**All patients *n* = 229**	**No infection *n* = 120**	**Any infection *n* = 109**	**OR**	**95% CI**	**Univariate *p*-value**
Sex, female	99 (43)	52 (43)	47 (43)	0.99	0.59-1.67	0.974
Age, years	71 [61–78]	72 [63–80]	68 [60–77]	0.99	0.97-1.01	0.152
Ethnicity, Caucasian	229 (100)	120 (100)	109 (100)			
**PRE-MEDICAL HISTORY**						
Hypertension	135 (59)	69 (58)	66 (61)	1.1	0.65–1.90	0.686
Diabetes mellitus	20 (9)	7 (6)	13 (12)	2.2	0.85–5.76	0.105
Oral anticoagulation	16 (7)	8 (7)	8 (7)	1.1	0.40–3.06	0.842
**ADMISSION NEUROLOGICAL AND CLINICAL FINDINGS**						
GCS Score	13 [7–15]	14 [10–15]	10 [3–14]	0.9	0.85–0.96	**0.001**
GCS Score <13	100 (44)	36 (30)	64 (59)	3.3	1.90–5.70	** <0.001**
ICH Scorexref^*^	2 [1–3]	1 [1–2]	2 [1–3]	1.6	1.25–1.90	** <0.001**
ICH Score >2	68 (30)	26 (22)	42 (39)	2.3	1.30–4.10	**0.006**
APACHE II Score	12 [9–20]	11 [8–17]	16 [10–21]	1.06	1.02–1.10	**0.004**
ICH volume, mL	21 [7–41]	18 [5–33]	24 [8–49]	1.02	1.01–1.03	**0.005**
ICH volume > 30 mL	81 (35)	33 (28)	48 (45)	2.1	1.20–3.70	**0.007**
Midline shift, yes/no	79 (34)	28 (23)	51 (47)	2.9	1.60–5.10	** <0.001**
Intraventricular hemorrhage	115 (50)	47 (39)	68 (62)	2.6	1.50–4.40	**0.001**
Infratentorial origin	21 (9)	9 (8)	12 (11)	1.6	0.63–3.86	0.338
**SURGICAL PROCEDURES AND HOSPITAL COMPLICATIONS**						
Mechanical ventilation	109 (48)	39 (33)	70 (65)	3.8	2.20–6.60	** <0.001**
Nasogastric tube feeding	107 (47)	33 (28)	74 (72)	6.7	3.70–12.10	** <0.001**
Hematoma evacuation	56 (24)	17 (14)	39 (36)	3.4	1.80–6.60	** <0.001**
Hydrocephalus requiring EVD placement	37 (16)	7 (6)	30 (29)	6.5	2.70–15.50	** <0.001**
Neurological deterioration^†^ (within 24 h)	84 (37)	31 (26)	53 (52)	3.0	1.70–5.20	** <0.001**
**OUTCOME**						
Length of NICU stay, days	8 [3–18]	5 [2–8]	17 [8–31]	3.8	2.90–5.10	** <0.001**
3-months mRS				4.8	2.50–9.50	** <0.001**
0	17 (7)	14 (12)	3 (3)			
1	29 (13)	23 (12)	6 (6)			
2	18 (8)	13 (11)	5 (5)			
3	25 (11)	14 (12)	11 (10)			
4	42(18)	19 (16)	23 (21)			
5	38 (17)	9 (8)	29 (27)			
6	60 (28)	28 (23)	32 (29)			

*All data given are median [IQR] or n (%). OR (95% CI) and univariate p-values represent associations between risk factors and any infections during ICU stay*.

*APACHE-II Score = the physiological subscore of the Acute Physiology and Chronic Health Evaluation; EVD = external ventricular drain; GCS Score = Glasgow Coma Scale Score; ICH = intracerebral hemorrhage; ICH Score = Intracerebral Hemorrhage Score; mRS = modified-Rankin-scale; NICU = neurocritical care unit*.

* *ICH Score (13) is a clinical grading scale to stratify the 30 days-mortality after ICH. It contains following variables: GCS Score (13–15 = 0; 5–12 = 1; 3–4 = 2), age, y (<80 = 0; ≥80 = 1), ICH volume, cm^3^ (<30 = 0; ≥30 = 1), the presence of intraventricular hemorrhage (IVH) (no = 0; yes = 1), infratentorial origin of ICH (no = 0; yes = 1). The higher the score gets, the more the mortality raises (range 0–6)*.

† *Neurological deterioration was defined as ≥2 points decrease in GCS or a new focal finding in a 24 h period after admission or postoperative. Bold values indicate statistically significant results*.

### Prevalence and Chronologic Course of Infectious Complications

**Figure 2:**
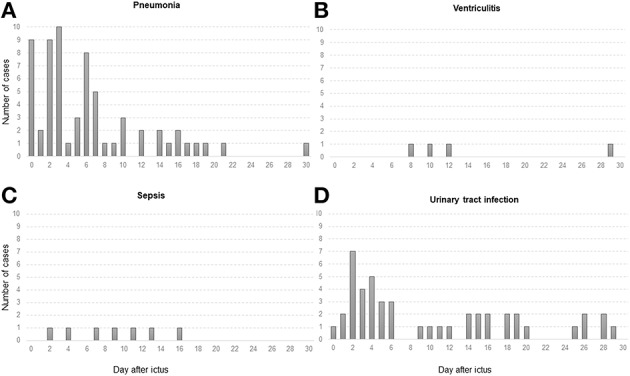
Graphs display absolute numbers of cases with infectious complications (y-axis) based on days after admission (x-axis) for **(A)** pneumonia, **(B)** ventriculitis, **(C)** sepsis, and **(D)** UTI (urinary tract infection). Pneumonia and UTI were mainly diagnosed in the first week after ictus (day 5 [IQR 2–9 days]; day 11 [IQR 3–25 days], respectively), whereas sepsis **(C)** and ventriculitis **(B)** occurred more frequently in the second week (day 11 [IQR 6–34 days]; day 11 [IQR 9–25 days], respectively).

**Figure 3:**
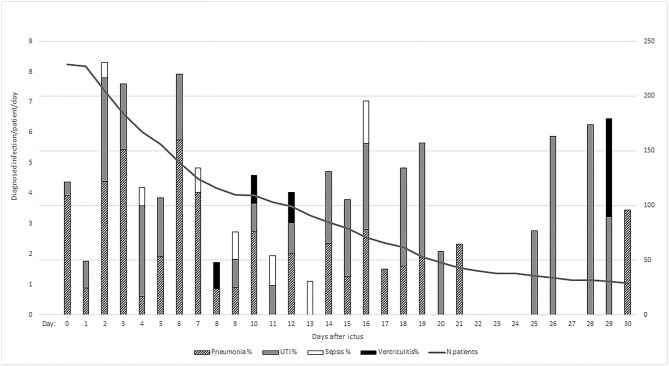
Represents relative prevalence of diagnosed infections per day/per patient to account for patients still admitted to the ICU. The absolute number of infections per day was divided by the number of patients per day. *N* patients, number of patients.

During NICU stay, a total of 109 (48%) patients developed at least one infection. The most common IC were pneumonia (*n* = 64; 28%), UTI (*n* = 54; 24%), sepsis (*n* = 9; 4%) and ventriculitis (*n* = 4; 2%) (Figures 2A–D). Thirty-one percent (*n* = 71) of patients developed one infection, 13% (*n* = 30) two, 3% (*n* = 7) three, and 0.4% (*n* = 1) of patients four. In non-mechanically ventilated patients, pneumonia was diagnosed in 10 (4%) patients within the early phase and in 8 (3%) patients in the late phase after ictus. IC showed an initial peak (≤48 h after admission) attributable to early-onset pneumonia (65%) and a second peak at days 6–7 [IQR 4–12 days], associated with VAP (76%). In addition, we found another peak around day 16 most likely associated with catheter-related infections and sepsis (Figure 3). In 42% (27/64) of patients with pneumonia a positive bacterial culture was obtained identifying gram-negative (18/27, 67%) and gram-positive rods (9/27, 33%). Patients diagnosed with UTI had corresponding positive bacterial cultures in 84% (45/54, gram-negative rods: 35/45, 78%, gram-positive rods: 10/45, 22%). In 50% (2/4) of patients with ventriculitis gram-positive rods were found. All patients with sepsis had a positive bacterial culture (9/9, gram-negative rods: 4/9, 44%, gram-positive rods: 5/9, 56%).

### Risk Factors for Infectious Complications

In univariate analysis, clinical and radiographic severity on admission, early neurological deterioration, and hospital-related procedures were significantly associated with any IC (Table 1). In multivariable analysis, early neurological deterioration (adjOR = 4.0, 95% CI 2.1–7.71, *p* < 0.001), hematoma evacuation (adjOR = 4.9, 95% CI 2.5–9.68, *p* < 0.001), mechanical ventilation (adjOR = 9.6, 95% CI 4.15–22.1, *p* < 0.001), nasogastric tube feeding (adjOR = 16.5, 95% CI 6.3–43.11, *p* < 0.001) and evidence of midline shift >4 mm (adjOR = 2.3, 95% CI 1.22–4.46, *p* = 0.011) were associated with a higher risk of pneumonia after adjusting for ICH Score. Twenty-four patients (11%) developed early-onset pneumonia, which was associated with later occurring sepsis (adjOR = 22.5, 95% CI 4.88–103.6, *p* < 0.001). In addition, nasogastric tube feeding (adjOR = 10.4, 95% CI 1.13–96.71, *p* = 0.039), neurological deterioration (adjOR = 13.8, 95% CI 1.65–115.7, *p* = 0.016) and mechanical ventilation (adjOR = 11.3, 95% CI 1.22–105.32, *p* = 0.033) were associated with sepsis. All patients diagnosed with ventriculitis had an external ventricular drain (EVD). None of the analyzed variables were significantly associated with UTI.

### ICH Score and Infectious Complications

**Figure 4:**
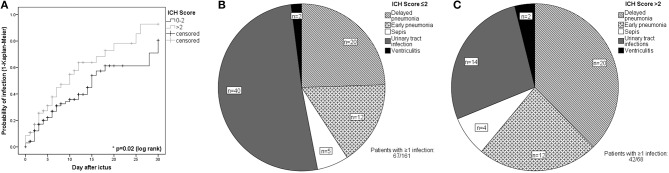
**(A)** Kaplan-Meier-Curve describing the probability of any infectious complication in patients with ICH Score ≤2 and >2. Patients were censored at the time of: withdrawal of care, NICU discharge, or death latest on day 30. Distribution of individual infections in patients with infections stratified by **(B)** an admission ICH Score ≤2 and **(C)** ICH Score >2.

The patients presented with a median ICH Score of 2 [IQR 1–3]. Patients with an ICH Score >2 were more likely to develop IC during NICU stay as compared to patients with an ICH Score ≤2 (40 vs. 32%, OR = 2.3, 95% CI 1.3–4.05, *p* = 0.006) (Table 3). In univariate analysis, patients with an ICH Score >2 developed IC earlier (Figure 4A). Moreover, patients with an ICH Score >2 were at higher risk for pneumonia (38 vs. 19%, OR = 3.6, 95% CI 1.9–6.6, *p* < 0.001) (Figures 4B,C).

### Infectious Complications and Outcome

Having at least one infection during NICU stay was associated with unfavorable 3-months outcome (adjOR = 3.0, 95% CI 1.41–6.54, *p* = 0.005). For each additional IC, patients had a 2-fold higher risk of poor outcome after 3 months (95% CI 1.16–3.57, *p* = 0.013) after adjusting for ICH Score and APACHE Score (Table 2). All ICH patients with sepsis had a 3-months mRS ≥3. Pneumonia was independently associated with poor functional outcome (adjOR = 4.2, 95% CI 1.33–13.19, *p* = 0.015).

**Table 2:** Risk for infectious complications for each point increase of the ICH Score.

	**OR**	**95% CI**	***p*-value**
ICH Score > 4	6.4	2.2–18.7	** <0.001**
ICH Score = 3	5.1	1.8–14.3	**0.002**
ICH Score = 2	4.3	1.7–11.0	**0.002**
ICH Score = 1	2.4	0.9–6.0	0.069

*ICH Score, intracerebral Hemorrhage Score*.

*Bold values indicate statistically significant results*.

**Table 3:** Risk factors for poor functional outcome (mRS > 2) evaluated in 229 ICH patients.

	**OR**	**95% CI**	***p*-value**
Number of infectious complications	2.0	1.2–3.6	**0.013**
ICH Score	2.2	1.4–3.4	** <0.001**
APACHE Score	1.1	1.01–1.2	**0.025**

*ICH = intracerebral hemorrhage; mRS = modified Rankin Scale Score; ICH Score = intracerebral Hemorrhage Score; APACHE-II Score = the physiological subscore of the Acute Physiology and Chronic Health Evaluation*.

*Bold values indicate statistically significant results*.

**Figure 5:**
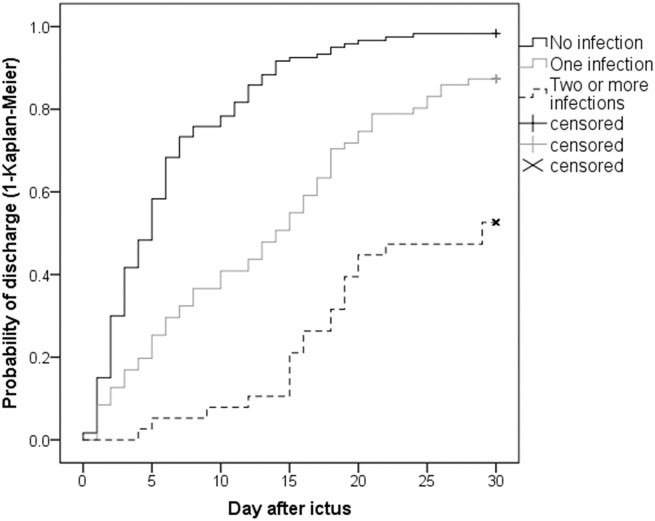
Length of NICU stay: Patients without any infection had a median LOS of 5 days [IQR 2–8 days], patients suffering from one infection 14 days [IQR 5–21 days], while patients suffering from two or more infections stayed 29 days [IQR 16–44 days] at NICU. All patients staying longer than 30 days were censored at day 30.

Moreover, the number of IC per patient was associated with the length of NICU stay (Figure 5): ICH patients with one infection were prone to prolonged LOS (adjOR = 1.4, 95% CI 1.01–1.82, *p* = 0.047) when compared to patients with no infection. This association was even more pronounced in patients with ≥2 infections (adjOR = 6.9, 95% CI 4.12–11.55, *p* < 0.001).

## Discussion

In the current study we found [1] that IC are common after ICH with pneumonia having the highest incidence, [2] that an admission ICH Score >2 may serve as a valuable early predictor for the development of IC during NICU stay, [3] that early-onset pneumonia may help to identify people at risk for sepsis at a later timepoint and, [4] that the number of IC is independently associated with prolonged hospitalization and poor 3-months functional outcome.

In our cohort, 48% of ICH-patients developed at least one infection during NICU stay, which is similar to previously reported prevalence rates ranging from 23 to 58% (7, 9, 10, 12). It is important to mention that our cohort includes all clinical and radiographic severity grades since all ICH patients presenting to our emergency room were included in the study. Pneumonia was the most common IC followed by UTI, sepsis and ventriculitis, comparable to formerly reported prevalence rates of 10–20%, 8–17%, 1–3.6%, and 0.3–3%, respectively (7, 11, 12, 20).

It remains important to identify patients at higher risk of IC already on admission to allocate more resources for infectious surveillance and the early start of preventive measures. We found that the ICH Score, assessed on the day of admission, may be a useful tool to identify these patients. The ICH Score is based on the GCS Score, age, ICH volume, the presence of intraventricular hemorrhage (IVH) and infratentorial origin of ICH, and has been introduced in the prediction of 30 days mortality after ICH (13). While a lower GCS Score, higher ICH volume, the presence of IVH, and older age as part of the ICH Score, have previously shown to be associated with the development of infections, the sum-score would be even more applicable for clinical practice. The major contributor to an increased ICH Score in our cohort was a lower GCS Score followed by ICH volume and the presence of IVH (data not shown). Therefore, the association of higher ICH Score and the development of infectious complications may simply be explained by the initial disease severity. Additionally, patients with IVH are at higher risk for the development of fever and more frequently require EVD placement (21) which may in addition contribute to the development of IC. This hypothesis is supported by the higher occurrence rate of ventriculitis (11%) in our patients with EVD, while no other risk factors for ventriculitis could be identified (22). Moreover, infratentorial hematoma location has previously been associated with neurogenic dysphagia (23) and may also lead to IC including the development of pneumonia (11).

Since we prospectively recorded the day of onset of each infectious complication, we found that patients with an early-onset pneumonia had 23-fold higher odds of developing sepsis at a later time point. Respiratory tract infections are the most common IC in intensive care units (24). The association with sepsis is of interest and may stratify patients at risk early, however this association has not been consistently reported in literature and may be attributable to the prolonged need for invasive catheters or decreased bowel movement with transition of gramnegative bacteria.

In the current study we mainly identified non-modifiable risk factors for the development of IC including admission disease severity, mechanical ventilation, surgical intervention and early neurologic deterioration, which is in accordance with previous studies (10, 25, 26). In hemorrhagic stroke patients it has been shown that early neurological deterioration is associated with higher rates of hospital complications and poor outcome (27, 28). While mechanical ventilation is a known risk factor for VAP (19, 20, 29), neurogenic dysphagia is common after ICH and may contribute to the development of aspiration pneumonia in non-intubated patients (30, 31). Another risk factor for aspiration pneumonia includes nasogastric tube feeding which can be reduced by early percutaneous endoscopic gastrostomy (32). Proposed interventions to reduce the occurence of pneumonia in mechanically ventilated patients include interruption of sedation, early mobilization, head elevation to 30–45°, oral care with chlorhexidine, retaining of ventilator circuits, selective digestive tract decontamination and prophylactic probiotics (33, 34). However, it is important to mention that in most of these studies patients with acute brain injury were not included. Our infectious disease prevention bundle encompasses the use of chlorhexidin, early mobilization and head-up positioning. Interruption of sedation was not routinely done in our poor-grade patients at risk for raised ICP (35).

The strong association between IC and 3-months outcome independently of co-morbidities quantified by the APACHE II Score in our patient cohort confirms recent studies, underlining the contribution of infections to higher NICU mortality and worse functional outcome in survivors (7, 12). In accordance with previous prospective observational studies in ischemic stroke and SAH patients (12, 29, 36), pneumonia and sepsis were independently associated with poor functional outcome. Limited by the low patient number with ventriculitis in our cohort, we could not statistically confirm the findings of former studies showing an independent association with increased mortality and morbidity (29).

The average NICU LOS was similar as previously reported in ICH patients [8 days] (9, 12). Likewise, the identified association between all types of IC and prolonged length of NICU stay is consistent with previously published data (9, 11, 12).

Our results have to be interpreted with caution due to their limited generalizability to all ICUs given that critical care management and in specific, the surveillance bundle for IC may differ. Moreover, all patients with the whole range of clinical grades are admitted to the ICU, which may not reflect clinical practices in other centers. Furthermore, we did not investigate the impact of central line placement and antimicrobial chemotherapy which may have influenced the occurrence and impact of IC. Another important limitation is the retrospective analysis of prospectively collected data, thus only associations and not causalities can be concluded. Finally, the aim of the study was not to find the most accurate multivariable model but to analyze whether the ICH Score would qualify for the prediction of infectious complications.

## Conclusion

Infections are frequent and serious in patients suffering from ICH. Their independent association with poor outcome warrants close surveillance and the earliest possible management when diagnosis is established. The association with prolonged NICU stay has a tremendous impact on the economic burden of this disease. Our findings may help to early identify patients at high risk for the development of IC.

## Data Availability

The datasets generated for this study are available on request to the corresponding author.

## Ethics Statement

All procedures performed in studies involving human participants were in accordance with the ethical standards of the Institutional Research Committee (Medical University Innsbruck, AN4088292/4.3) and with the 1964 Helsinki declaration and its later amendments or comparable ethical standards. Written informed consent was obtained from all patients or legal representatives according to local regulatories.

## Author Contributions

AL and RH study idea and design, data acquisition and analysis, manuscript writing and drafting, MK, VR, BI, MG, AS, RB, SL, PR, BP, ES, and CT contributed to the study idea, data acquisition, manuscript drafting. RH contributed to the study supervision. All authors read and approved this version of the final manuscript.

### Conflict of Interest Statement

The authors declare that the research was conducted in the absence of any commercial or financial relationships that could be construed as a potential conflict of interest.
